# What interferes with conducting free lists? A comparative ethnobotanical experiment

**DOI:** 10.1186/s13002-021-00432-5

**Published:** 2021-01-23

**Authors:** Melise Pessôa Araujo Meireles, Ulysses Paulino de Albuquerque, Patrícia Muniz de Medeiros

**Affiliations:** 1 Licenciatura em Educação do Campo, Universidade Federal do Piauí- Campus Senador Helvídio Nunes de Barros, Piauí, PI 6460-7670 Brazil; 2 Departamento de Botânica, Universidade Federal de Pernambuco, Recife, PE 50670-901 Brazil; 3 Centro de Ciências Agrárias, Universidade Federal de Alagoas, Rio Largo, AL 57100-000 Brazil

**Keywords:** Data collection, Ethnobiology, Ethnobotany, Medicinal plants, Ethnobiological methods

## Abstract

**Background:**

The free list, also written “freelist”, or “free recall”, is an ethnographic method that characterizes the local knowledge of a population about a given cultural domain. However, there is still much to elucidate about the variables that can influence the number of items that participants cite using this technique. This study applied a casual-comparative experimental design to analyze whether 3 months’ time, age, and external stimuli influence the similarity of plant free lists applied at different times.

**Methods:**

Data was collected from 103 farmers from the rural community Alto dos Canutos, in the municipality of Picos, Piauí state, Brazil. Two free lists were conducted at two different times, with an interval of three months between them. Subsequently, the similarity between the first and second free lists of each participant was calculated using the Jaccard Similarity Index. The generalized linear model (GLM) with binomial errors and stepwise approach was used to analyze the effects of age and external stimuli on information collection when comparing free lists applied at different times.

**Results:**

Participants’ age influenced the information that the free lists collected, demonstrating that the older the participants, the lower the similarity among the free lists. Among the external stimuli analyzed, only the presence of third parties influenced the content of the free lists at the time of the interview. However, contrary to expectations, third-party presence positively influenced the similarity of the lists.

**Conclusion:**

The results show that the studied variables age and third-party presence can influence the capture of knowledge. These findings warrant future research into the influences’ causes and their potential mitigation, e.g., by isolation or by breaking the medicinal plant domain into focused sub-domains and conducting simpler, successive free-lists, which can mitigate memory issues.

**Supplementary Information:**

The online version contains supplementary material available at 10.1186/s13002-021-00432-5.

## Background

The free list is an ethnographic method used to list elements of a given cultural domain [1, 2], whereas cultural domain is understood as the set of cultural information belonging to the same category [3]. It is widely used in ethnobiological research to list domain elements, in the order in which they come to the mind of the interviewee. Items are identified when the interviewee is asked to list everything they know that is in “X”, the “X” being the name of the domain to be questioned [4]. An appropriate question for using the free list to identify information in a category may be, for example: “which medicinal plants do you know for treating wounds?

The elements captured from this question reveal information both about the items mentioned, as well as about the people who listed them, showing remarkable data in a community/culture [5], such as the importance of items based on the community’s consensus (frequency of convergent reports) [6]; the local preference for components used during the interview period of time [7]; the determination of local specialists, since large lists characterize the most knowledgeable people [6], and the listing order of the items, in which those an individual mentions first are cognitively prominent or “salient” in relation to the last ones [8]. Items’ order in lists, together with the number of times the item is cited, serves to calculate the Salience Index [9, 10] that determines the cultural relevance of a term or element. However, some studies use the free list without taking the Salience Index into account in their analyses and only draw items based on the cross-list frequency of the item’s mentions [11–14].

In many cases, an interviewee may not remember an item she/he knows during the interview [1, 6, 15], which brings limitations to the free lists’ responses.

Ethnobotanists also employ free lists to compare individual knowledge (intracultural comparison), for example, by evaluating the influence of gender and age in the number of cited items. Such application of the technique is being increasingly used in the last decades. Thus, it is possible that individual knowledge capture may be incomplete, which may have consequences on the research results, since the hypotheses may be confirmed or disproved based on responses that are biased by different factors.

To understand what influences participants’ responses while collecting of ethnobiological data, it is essential to consider age because the literature has offered evidence of its influence on inventories. This is evidenced, for example, when capturing information about plant species, since memory lapses are more frequent in the elderly [16]. Conversely, there are works that report elderly people as specialists and protectors of local knowledge [17], which shows they know a greater diversity of ethnospecies [18, 19] and their applications [18, 20]. However, other studies have not found great differences in the knowledge of plants among the oldest and youngest [16, 21]. Thus, it is often not possible to know if this lack of differences is concrete, as it could be related to a memory lapse effect that more affects the elderly during the interviews.

While certain variables are believed to affect collection of information, the approaches taken so far to analyze age interference only ascertain knowledge levels by comparing information obtained from the various members of a researched group. It is necessary to assess the effects of extrinsic variables on the technique by analyzing data collected from the same individual at different times.

Besides age, ethnobiological research mentions other variables as possible causes of interference during knowledge capture, such as the location and presence of third parties [22]. Miranda et al. [23] provide evidence that different interview locations (e.g., fairs, supermarkets, plant stores, and a control location) may provide lists with different sizes and repertoires of plants. However, it remains necessary to discover whether interviews conducted in closed and related places, such as the environment that makes up a community, may or may not lead to free lists with different characteristics.

The presence of third parties, during an interview, may be subject to biasing the data in some way, such as changes in participant’s responses [22] or by third parties interfering by offering suggestions [6]. These changes in responses were confirmed in works developed by Boeji [24] and Aquilino [25], when researching marriage. However, although they were important to show the potential biases deriving from the presence of third parties, these studies were not ethnobiological. Thus, it is necessary to assess how much third party presence, simple or with interference, actually influences the content of free lists in ethnobiological research.

Ethnobiological research has been concerned with testing hypotheses [26]. Therefore, it is necessary to understand the way data is collected. This paper does not propose to prove whether the free list is an adequate data collection technique or not, but instead to analyze which situations benefit or impair its application.

The free list was the technique chosen among other data collection techniques, such as the checklist, as the free list avoids leading or cuing interviewee and makes subjects rely more heavily on memory. The checklist, on the other hand, offers the plant as a visual stimulus so that a person remembers specific uses. However, for other data collection techniques with structures that are similar to free lists (starting from the category of use or general uses and requesting the plants/use/parts used), it is believed that their behavior would be similar.

Therefore, here we analyzed the following hypotheses: (1) the older the person, the lower the similarity between the free lists of plants mentioned at different times; (2) external stimuli (location of the interview, presence of third parties, interference from third parties and other factors that may influence the interviews) have an influence on the similarity of content of free lists conducted at different times, and (3) the most cited plants remain the same in different free list moments. Then, given the importance of the variables that can affect free list results in ethnobiological research, free lists conducted during different events were compared. Although we analyzed aggregate data to test hypothesis 3, our main focus was on the use of free lists to evaluate individual knowledge. We are aware that this is not the most widespread use of the technique, and that the theoretical base of the free list is associated with the understanding of the cultural domain, but our choice is justified given that (1) several recent ethnobiological studies are focusing on individual differences in medicinal plant use, and (2) the usefulness of aggregate free list data has been widely discussed in ethnobiological literature (see, for example, [27]).

## Material and methods

### Description of the study area

The study was carried out in the state of Piauí, in the municipality of Picos (7°04′37′′ S and 41°28′01′′ W). This municipality has approximately 78,222 inhabitants [28] and is located on the right bank of the Guaribas River, integrating 1 of the 39 municipalities that make up the Guaribas Valley. Inserted in the Southeast mesoregion of Piauí, it has an area of 803 km^2^ and is limited to the municipalities of Santana do Piauí and Sussuapara to the North, Itainópolis to the South, Dom Expedito Lopes and Paquetá to the West, and Sussuapara and Geminiano to the East. The municipality is 306 km away from the capital Teresina [29].

The climate, according to the Köppen classification, is of the BSh type—hot and semi-arid with summer rain and poor rainfall during most of the year [30]. The soils have a geological alteration of the sandstones, siltstones, and shales, and present red-yellow argisol soils, and Neossolos soils considered essentially quartzous, deep, drained with low fertility, and with plant transitions. Vegetation includes deciduous forest and sub-deciduous hyperxerophilous caatinga phase and/or sub-deciduous savanna/sub-deciduous forest [31].

**Fig. 1:**
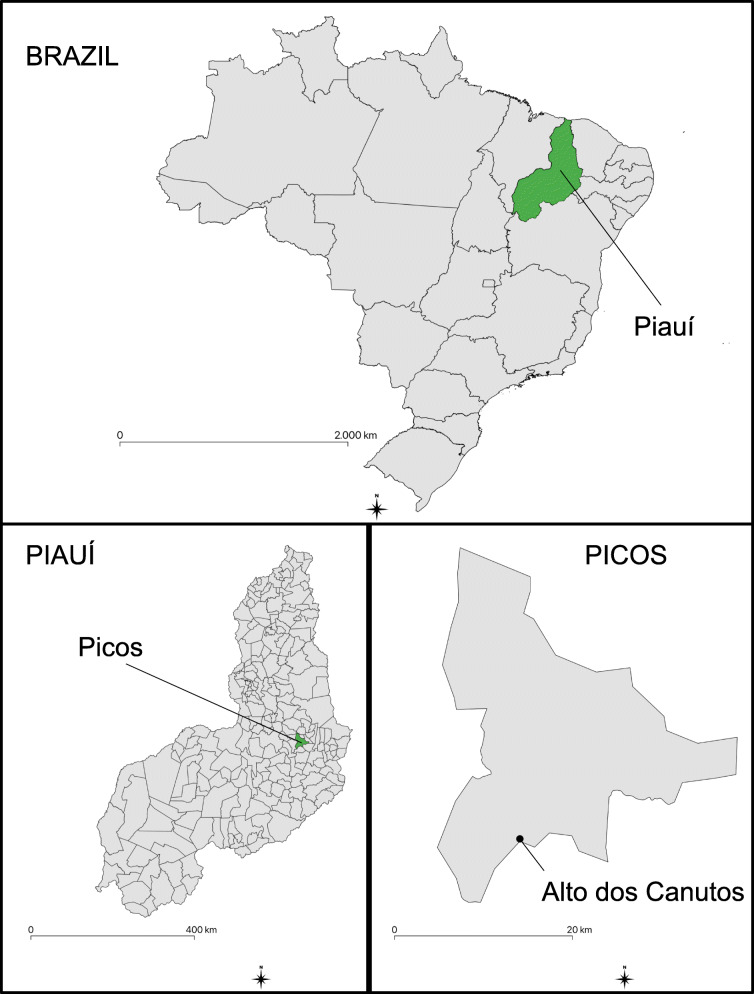
Location of the Alto dos Canutos community, located in the municipality of Picos, Piauí, northeastern Brazil

The rural community Altos dos Canutos (7°09′51′′ S and 41°33′5′′ W), which is located 14 km from the center of Picos, was chosen for this study because it is a representative rural community of the region, with easy access to the residence of all inhabitants, and because it has a local plant-dependent medical system (Fig. 1).

The community has 214 inhabitants distributed among 62 households, according to data provided by the agent of the Family Health Post that exists in the neighboring community (Vigia Community). Its economic activity is based on family farming, with the cultivation of beans (*Phaseolus Vulgaris* L.) and corn (*Zea mays* L.). The families consume their production while the surplus is traded.

Only the recently built main street of the community is paved. All houses are made of masonry and have running water, electricity, and regular, weekly garbage collection. The community has a Health Post, where the doctor visits once a week. The Community’s nursery school and elementary school have been deactivated, and the students transferred to the village of Torrões, located 9 km away from the focal community. Students now commute to school by bus.

### Community recognition and ethical aspects

Before establishing contact with the community, authorization was obtained from the Picos Rural Workers Union. Subsequently, the initial communication with the community was facilitated due to the assistance given by the representatives of the Community Residents’ Association who promoted a meeting to present the research and the researchers.

The research was submitted to the Research Ethics Council (CEP) of the Federal University of Piauí and approved under opinion number 89553018.5.0000.8057. In compliance with the precepts of the National Health Council (Resolution n°466, December 12, 2012), which oversees ethical aspects of research involving human beings, the objectives of the research were explained to community members. Those who agreed to participate showed permission by signing the Free and Informed Consent Form (TCLE) and through their individual authorization to enter their residence.

### Interview data

The community has 62 households with 214 residents. As the research was restricted to household providers, a total of 124 residents were interviewed. However, as some residents are widowed, separated, or live alone, the initial target of 114 residents were invited to participate.

The potential sample of 114 participants was impossible by some residents declining to collaborate in the research, others were not in their homes during the research execution and others had health problems, which limited their interview contributions. The collection of ethnobotanical data was thus attended by a sample of 103 residents.

The interview took place in two stages: in the first stage, data on the profile of the participants were collected. The free list [4] was applied subsequently, which had the generating question: what medicinal plants do you know? Then, the interviewees were encouraged to link the plant mentioned with the therapeutic indications, part of plants used, mode of use, whether the plant was used, and whether it had any contraindications. Three months after the first round of free lists, the second stage began, in which the free list application was repeated using the same interviewees and the same questions, with the aim to compare the two sequences.

The 3-month period of this survey was a decision made respecting the logistics of the work to make sure that all residents were interviewed in the same period in both stages. In addition, this interval was proposed so that it would not become tiring for the participants, since the lists were applied to the same interviewee using the same questions.

The following protocols were used to conduct the interviews:

1. All interviews took place with the same interviewer;
2. There was a difference of 3 months from one interview to another for all participants;
3. The participants were informed that the interviews should be carried out individually, avoiding other people nearby. However, this was not always possible, as there were times when other people appeared during the interview. The fact that a third person remained in the same room as the interviewer and interviewee during the interview was considered as presence of third party and interference by third party if this person at any time intervened (trying to assist the informant);
4. Participants were approached at their homes. There were some cases where the participants were found at the neighbors’ house or working next to their residence and asked to be interviewed in these places; thus, two types of environments were obtained: closed space, inside residences, and open space, in which the interview took place outside the residence, such as the footpath or the balcony of the house;
5. Priority was given to the place where the participant felt most comfortable and away from areas of greatest activity. However, some participants performed certain activities at the time of the interview. Factors we considered as potential interview interference included the participant having the television on, using the cell phone or simultaneously performing some domestic activity. Unforeseen factors that could also influence were recorded during the interview.
6. The participant was informed that the interviewer would record their response in writing.

During the application of the free lists, the observation approach [32] was used, which allowed the interviewer to be aware of the possible variations that could occur and to record information in a discreet way. For this, the interview forms had fields to record the presence of third parties, their degree of kinship with the interviewee, whether this presence caused any intervention, the place where the interview was conducted, and other possible factors that could interfere.

To encourage informants’ memory and to increase the efficiency of the free lists, non-specific induction, reading of items and semantic suggestions were used in both stages. Non-specific induction consists of asking the interviewee for the second time, for example: what other plant do you know as medicinal? A rereading entails the researcher say the items already mentioned during the interview. During a semantic suggestion, the interviewer asks what other items can be mentioned that are similar to those already mentioned [33]. This technique allows the participant to remember items that they did not mention that can still be added to the free list.

Regarding the profile of the interviewees, the age group of the interviewees ranged from 21 to 85 years with 33% being young adults (aged from 21 to 39 years); 36.9% middle aged adults (from 40 to 59 years) and 30.1% elders (aged from 60 to 83 years). The number of species cited in the first free list event reached 116 species, 25 of which were exclusive to this moment. The second free list event reunited 100 plants, 11 being exclusive. Altogether, the community cited 127 different species of medicinal plants (Table 1 -Additional file 1).

### Botanical material collection

After the end of each free list, the plants cited were collected, giving preference to fertile specimens following the procedures used in the studies of plant taxonomy [34]. However, some plants were not found in the native vegetation of the community, as they were bought in the market or in a medicinal plant fair, in the municipality of Picos-PI.

Herbalization techniques were applied to the collected plants for exsiccates and duplicates, according to the usual procedure [35] and the voucher specimens were deposited in the Herbarium Delta do Parnaíba (HDELTA), placed in the Universidade Federal do Delta da Parnaíba (state of Piaui).

Botanical identifications were made with reference in the specialized literature and by consultation by a specialist. The classification system adopted was the Angiosperm Phylogeny Group IV [36]. The Latin names were revised and the abbreviations of the authorities names acquired through the Missouri Botanical Garden database [37].

### Preparation and analysis of ethnobotanical data

A binary (presence and absence) matrix was entered in excel with the registration of all species cited in the participant’s two free lists for the preparation of the data.

The two free lists were compared for each participant. We indentified (a) the plants that were cited in both free lists, and (b) the plants that were cited in only one of the free lists. From these data, the similarity of the plants cited was calculated using the Jaccard Similarity Index (JI) [38] for each participant, through the following formula: JI = a/a + b. From the data similarity results obtained from each participant, the mean and standard deviation were calculated. When compared, the free lists showed low similarity with a mean of 0.26 and a standard deviation of 0.16.

The generalized linear model (GLM) with binomial errors was used to test the first and second hypotheses. At first, the explanatory variables were age, place of interview, presence of third parties, interference by third parties during the interview and presence of factors that could affect the interview. However, a Farrar test of chi-square, using the function ‘omcdiag’ of the package ‘mctest’ of R, revealed multicollinearity. Thus, we removed the third party interference variable from the model. The response variable was a double column with (1) the number of double presence of the species mentioned in the two free lists, and (2) the number of plants exclusive to a single list (associated columns using the “cbind” function of R). The stepwise approach was also used to leave the variables that gave the lowest AIC values in the model (Akaike Information Criterion). The generalized linear model (GLM) with binomial errors was performed using Software R (version 3.5.0) [39]. Except for age, the other explanatory variables were coded for use in GLM as explained below:

1. The location was assigned 1 if it was the same in both moments of the interview and 0 if it was different;
2. The presence of third parties was assigned the value 0, if no one was present at the different moments of the interview; 1, when someone was present at one of the moments of the interview; and 2, when there was the presence of a third party at both moments of the interview.
3. Third party interference was assigned the value 0, if it had not interfered or if there was no third person present during the interview; 1, if a third person had interfered in one of the free lists; and 2, if there was interference in the two free lists.
4. About the other factors that could influence the interview (called “presence of factors”) these were assigned the value 0, if not present at any time during the interview; 1, if the interference factors were present in at least one of the moments of the interview; and 2, when these factors were present in both moments of the interview.

The scenarios where the interviews took place and the variables considered as an external stimulus are described below (Table 1):

**Table 1:** Description of the scenario in which the interviews took place and the variables considered as external stimulus of the Altos dos Canutos Community, Municipality of Picos, PI

Variables	Percentage (%)
Location	
Same location	61.16
Different location	38.84
Location type	
Interviewee’s house	91.26
Neighbor’s house, work	8.74
Open place	57.28
Closed place	42.71
Third party presence	
Absence	23.31
One event	54.36
Two events	22.33
Third party	
Friend	6.77
Family (grandchild, uncle, mother in law, father, etc.)	42.37
Partner	42.37
Neighbor	8.47
Third party interference	
No interference	33.01
Interfered in one free list	55.34
Interference in the two free lists	11.65
Presence of factors	
Absence	63.10
One event	33.98
Two events	2.92
Type of factors	
Watching TV	26.82
Working	19.52
Domestic activity	19.52
People chatting nearby	12.19
Caring for child	14.64
Others	7.31

To test the third hypothesis, we performed a Spearman correlation test with the number of interviewees that mentioned each species in the two free list events

## Results

### Does the age of the interviewee influence on the recall of medicinal plants known to them?

It was confirmed that age was one of the variables that made up the model, so that older people showed less similarity between their lists, reducing the double presence of the species mentioned in the free lists compared to other age groups (Table 2).

**Table 2:** Summary of the generalized linear model followed by stepwise for the effect of the variables age and presence of third parties on the double presence of species mentioned in the two free lists in relation to the single presence by residents of Altos dos Canutos Community, municipality of Picos, PI

	Estimate Std.	Error	*z* value	Pr(>|z|)
(Intercept)	− 0.520364	0.192894	− 2.698	0.00698 **
Age	− 0.007061	0.003246	− 2.175	0.02962 *
Third party presence	0.171101	0.080250	2.132	0.03300 *

Signif. codes: 0 ‘***’ 0.001 ‘**’ 0.01 ‘*’ 0.05 ‘.’ 0.1 ‘ ’ 1

AIC: 434.1

Residual Deviance: 141.5

### Do external stimuli influence the content of free lists?

The location where the interview was conducted, the interference of third parties and other potential interview-influencing factors did not remain in the model, as they did not interfere with the free list content during data collection. Still, contrary to expectations, the mere presence of third parties positively influenced the free listing responses (Table 2), making the free lists more similar.

### Were the most cited species the same in the two distinct free list events?

We found a strong and positive correlation between the species’ citations in the two free list events (rs = 0.79; *p* < 0.001). The top 3 most cited species were the same in both moments. They were *Mentha × vilosa* Huds. (*n*_1_ = 46 and *n*_2_ = 48), *Amburana cearensis* (Alemão.) A.C. Sm. (*n*_1_ = 40 and *n*_2_ = 43) and *Lippia alba* (Mill.) N. E.Br. ex. Britton and P. Wilson (*n*_1_ = 39 and *n*_2_ = 43).

## Discussion

### Does the age of the interviewee influence the recall of medicinal plants known to him?

The data obtained for this research show that older people had less similar free lists, thus reducing the double presence of species between the lists. This indicates that even in the face of a relatively short interval (3 months), as proposed in this research, elderly people may have trouble efficiently recalling the names of plants they know.

In ethnobiological research, age is identified as a factor that influences traditional botanical knowledge [40]. When comparing the data obtained for this research with that of the literature, there is a disparity of results in relation to studies that report on the influence of age on knowledge about medicinal plants. Studies point out that it would be best to collect data from older people, as they generally have greater knowledge about natural resources. Thus, they are considered to have knowledge about medicinal plants, both for their ability to retain it throughout life, derived from lived experience [41], and due to the frequent use they make of them [6], which is related to the number of plants that are cited in a free list, for example. These works end up restricting comparisons between individuals of different generations [41–43].

It is important to note that this study compared the similarity of cited species and as there was a time interval from one free list to another, it is necessary to consider that the difference between the lists may be due to something temporary in these individuals. Even with this observation, the result of this research indicates the need for greater caution regarding the collection and analysis of information from this individual knowledge, especially when related to this age group, in order to avoid bias in the results.

A possible justification for presenting different free lists in this study is due to a characteristic inherent to older people: impaired memory. Some researches claim that elderly people are more forgetful than young people [18, 44] and attribute this forgetfulness to findings in which recorded knowledge begins to decline after the group aged between 59 to 68 years [18]. Thus, the interviewees of this group do not remember all the medicinal plants they know, needing more effort to remember, which brings limitations in the answers.

In these cases, it cannot be affirmed precisely that a greater number of plants mentioned by one participant represents greater knowledge in relation to another participant who mentioned fewer plants. Thus, in communities with elders, it is necessary to avoid evaluations that consider individual knowledge, based only on the number of plants mentioned in a single free list.

It is suggested the use of more than one free list, with the same participants, in communities with high rates of elderly people. This will help to collect more complete data about plants known to this age group and will help to validate individual knowledge, thereby improving the quality of the data collected. Another suggestion would be to opt for the interview-checklist [45], a technique that provides more stimulus to the interviewee and provides less memory effort in relation to the free list. In addition, it allows the interviewee to be more comfortable and makes it easier to remember the question. Wiryono et al. [44] recorded the correlation between botanical knowledge and people’s age. The authors observed that when using photographs to identify known plants within a community, the elderly recognize more plants and better report their respective uses in relation to younger people. However, there is still a few studies that link age and the use of visual stimuli, requiring further testing for this statement.

In view of the context presented above on the influence of age on the collection of ethnobiological data, the researcher who is aware of this approach will be able to identify the technique that best suits their research, and it is up to them to select the best data collection instrument to capture the desired information.

### Do external stimuli influence the content of free lists?

Regarding the different places where the interviews were conducted, our results show that it did not interfere with the plants cited. In contrast, the study by Miranda et al. [23] reported that the place where the interview is conducted has a direct influence on the responses mentioned by the interviewees, in which the participants listed the plants according to the local context in which the interviews were conducted. Thus, ethnobiological studies suggest that the presence of environmental stimuli during the execution of free lists presents a limitation to the method, as the participants can be influenced by them at the time of the interview.

As in the present study, where the interviews were conducted with non-specialists and with native vegetation occurring close to the residences, the interview location was also studied in southeastern Madagascar. That study found that the proximity of the participants’ house to the forest had no influence on the medicinal plants mentioned [12]. In this rural Brazilian community, interviews were conducted in closed and in open spaces, where in the latter the participant had visual contact with nature and yet location did not interfere with list’s content of the free lists. However, studies need to be repeated in other communities to understand if these results represent a general pattern.

In ethnobiological research, interviews can take place in various environments, such as, at the participant’s home, close to home, at the workplace, among others and in living spaces. Among those, the living space is the least suitable in opposition to the other places, where the participants have a greater control of the spaces [22] and feel more comfortable. In addition, a quiet place, away from the areas of greatest activities should be prioritized [46]. In the studied community, the interviews were conducted at the participant’s house, the neighbor’s house and at the workplace (within the community), a very familiar environment and perhaps that is the reason the place has not influenced on the collection of information, as the selected space prepares a relaxed and enjoyable environment for the interview.

Regarding the presence and interference of third parties, studies with married couples have found that the presence of the spouse affected participant’s responses, either because the partner’s presence brought positive evaluations about the marriage or because a spouse had to agree with the partner, making disclosure of negative or conflicting positions difficult [47, 48]. It is recommended that studies aimed at assessing individual knowledge conduct one-to-one interviews. Yet, there are still no ethnobiological studies that address the influence of a third-party presence or interference of a third person in ethnobiological studies.

Other studies suggest that the presence of third parties may interfere in the results. This may happen at the moment when responses are suggested to the interviewee, impairing the data collection, since the items must be said by the interviewee in the order that comes to their mind [6]. Thus, the presence of a third person should be avoided [46], although sometimes it is not possible to prevent it. In this study, it was found that the interference of third parties did not influence on the collection of information.

The existence of a third person had an opposite effect to the expected, positively influencing the similarity of the two lists obtained, increasing the double presence of items and allowing the participants to remember the same plants reported in both free lists. This demonstrates that despite the fact the researcher is a stranger in the community, there is the possibility of causing a certain discomfort and shyness during the interview, which could exert influence on the free list. However, the presence of a known person (friend or relative) during the interviews was positive and made the participant feel more comfortable, making the family environment pleasant and safe to mention what they know about medicinal plants. The presence of another person during the interview may have caused the interviewee to remember some items due to shared experiences about medicinal plants with this person previously.

This may also be related to the fact that the interview on medicinal plants does not have questions that are too intimate or sensitive to the interviewee to become embarrassing in the presence of another person. Thus, it is believed that the presence of a third party interferes with the interview only when they are interested in the interviewee’s response [25, 47], and reporting on known medicinal plants within the community is a relatively simple question.

For example, people may feel intimidated in reporting about some plants, especially if they are related to something more intimate, such as diseases related to the genitourinary system (prostate, urinary inflammation, inflammation of the uterus, among others), and this can lead the participant to omit certain plants. However, it is worth mentioning that the question was very general: which medicinal plants do you “know”? Thus, they were able to cite the plants without directly relating to those that are used in their daily lives. This possibly did not interfere with the fact that a person was present during the interview.

In this context, the researcher will be aware of his actions when using this technique in the presence of third parties. The researcher may, depending on the circumstances, decide whether to continue the interview, return at another time and/or simply leave. Thus, the presence of a third party can be beneficial if the objective of the research is to capture the general knowledge of a community.

However, we stress the need for more detailed research, in order to verify in which situation the presence of a third party is positive or negative during the interviews with the use of the free list. For example, checking whether the presence of a person of the opposite sex intimidates the participant from reporting certain types of plants associated with the treatment of any gender-related illness.

Even though it is not the interviewer’s desire, the interviews suffer external influences during the research, which is beyond the control of the researcher [46]. During this work, “potential interview-influencing factors” (Table 1) were also present, although these did not interfere with the plant species mentioned in the free lists.

The low similarity found in the free lists at different times indicates that something made the participants lose their attention during the interview. However, extrinsic factors were not the drivers of such inattention. We therefore, believe that these factors have not interfered free lists similarity precisely because they make the environment as natural as possible for the interviewee. If the interviewees had been placed in an artificial context, that is, in a controlled interview location, it could have been difficult to report the information. This finding is consistent with the literature [49], which states that for data collection to occur effectively it is necessary that it takes place in the most familiar way to the interviewee.

### What else could influence the similarity of free lists?

Considering that extrinsic factors were not able to explain free lists similarity, perhaps intrinsic characteristics of the free lists account for this behavior. Free listing has the reputation of being a simple data collection technique that allows information to be obtained quickly [6], only requiring short answers, which would decrease the chance of the participant not concentrating on answering them [50]. However, the plant medicinal category is often a large domain. Kujawska et al. [51], for example, studied rural communities in Misiones (Argentina) and found that the medicinal category presented the highest number of species and properties. Therefore, people trying to list all medicinal plants are essentially juggling several (sub)categories in their minds, which is much more taxing to the mind’s executive functioning. For this reason, some researchers have argued that narrowing the domain could make the free lists more complete whereas general domains result in incomplete and scattered lists [27]. The difficulties in successfully mentioning items within a large domain can be due to memory issues (as discussed before) and potentialized inatention in contexts of long-lasting interviews. For this reason, in situations when the elders are the most knowledgeable, their high number of known species can increase the odds for information omission during the free lists. This pattern may also have contributed to the lower free list similarities for the elders.

### Were the most cited species the same in the two distinct free list events?

Although some medicinal plants were only mentioned in one of the free list events, different free lists tend to indicate the same species as the most cited. Although free lists focus on individual knowledge, each individual is part of a higher instance (i.e., local knowledge) [52, 53]. Therefore, individual omissions do not seem to bias the free list aggregate outcomes, which is why it is a (well-known) important tool to search for culturally important species.

However, as most ethnobiological studies with free lists focus on the whole set of medicinal products (plants and/or animals), it is possible that interviewees disproportionally forget/omit certain species or subcategories. Flores and Quinlan [54] investigated medicinal plants used by Dominicans and found that the free lists did not exhibit the gynecological conditions for which they used medicinal plants. For this reason, we need to study the effects of different data collection tools on the aggregate data outcomes as well as the effects of narrowing the domain (e.g., focusing on specific therapeutic indications).

## Conclusion and future perspectives

To understand the best practices for free list use, this research highlighted some free-listing variables. To cover all of them, a generalist approach was applied. The results analyzed can serve as a basis for future studies, even though we know that there are other variables in this scenario that also deserve to be analyzed, such as the socioeconomic profile and education, to understand how they affect the collection of ethnobiological data. In addition to these, it is also necessary to investigate the environmental context in which the informant is inserted in relation to variations in the environment and the availability of plant resources. This could be achieved by raising the following question: will the plants present in the surroundings of a community be present in people’s memory even when they are in a closed environment, then being remembered?

Our results support the use of free lists for evaluating aggregate outcomes (e.g., the identification of culturally salient species). However, its more recent applications for intracultural comparisons may bring some biases to ethnobiological research.

The age factor influenced the data collection process during the free lists’ execution. The interviewer, knowing the consequence that the free list has, can choose whether or not to use this data collection technique to capture the desired dimension. If this technique is selected, our suggestion is that it is performed twice with the same sample to access more data, check the difference between the two moments, and thus be able to validate the knowledge and increase the reliability of the data collected by people within this age range. Narrowing the research domain can be a good solution to make an adequate use of free lists. Memory and inattention biases may me much lower for short interviews with less response options.

Regarding the presence of third parties, this study showed a positive influence that caused the interviews to present more similar lists. With this, it is believed that the interviewer does not always need to worry about the presence of third parties, being able to decide whether or not they remain during the application of a free list. Therefore, if the researcher’s objective is to recover the maximum knowledge of plants disseminated within the group of people being studied, the researcher need not have concerns about the presence of third parties. However, this presence needs to be rethought if the aim is to understand the participant's individual knowledge. Further investigations are still needed to verify in which situation the presence of a third party is positive or negative during the interviews with the use of the free list, for example, to verify if the presence of a person of the opposite sex intimidates the participant to report certain types of plants associated with some gender-related diseases.

## Supplementary Information

**Additional file 1: Table 1.** Plants mentioned in the two free lists, method of preparation, function and parts used by residents of the Alto dos Canutos community, Picos, PI.

## Data Availability

We have already included all data in this manuscript
